# The Long Noncoding RNA LINC02820 Promotes Tumor Growth and Metastasis Through Regulating MYH9 Expression in Esophageal Squamous Cell Carcinoma

**DOI:** 10.1002/mco2.70218

**Published:** 2025-05-23

**Authors:** Xiaomin Xu, Xinting Mao, Wenrong Liu, Yue Ming, Tingting Zhang, Yang Yang, A‐Lai Gu‐Ha, Yi‐Dan Lin, Yong Peng

**Affiliations:** ^1^ Center for Molecular Oncology Frontiers Science Center for Disease‐Related Molecular Network State Key Laboratory of Biotherapy and Cancer Center West China Hospital Sichuan University Chengdu China; ^2^ Department of Thoracic Surgery West China Hospital Sichuan University Chengdu China

**Keywords:** esophageal squamous cell carcinoma, LINC02820, MYH9, IGF2BP2

## Abstract

Long noncoding RNAs (lncRNAs) play important roles in tumorigenesis, but their biological functions and mechanisms in esophageal squamous cell carcinoma (ESCC) remain poorly understood. In this study, we employed high‐throughput sequencing and bioinformatics analyses to identify the differentially expressed lncRNAs between ESCC tumors and adjacent normal tissues, among which LINC02820 is significantly upregulated in ESCC. Rapid amplification of cDNA ends assays determined the transcription initiation and termination sites of LINC02820, confirming it as a novel transcript variant localized in both the nucleus and cytoplasm of ESCC cells. Functional studies demonstrated that LINC02820 promotes cell proliferation and migration in vitro and enhances tumor growth and metastasis in vivo. Mechanistically, LINC02820 interacts with Myosin‐9 protein and prevent it from ubiquitination‐mediated proteasomal degradation. Additionally, the RNA‐binding protein insulin‐like growth factor 2 mRNA‐binding protein 2 binds to LINC02820 and increase its RNA stability in ESCC cells, thus upregulating LINC02820 expression. Therefore, these findings indicate LINC02820 as an oncogenic lncRNA in ESCC progression and suggest its potential as a therapeutic target.

## Graphical abstract

This study demonstrates that the RNA‐binding protein IGF2BP2 binds to and stabilizes LINC02820, leading to elevated LINC02820 expression in esophageal squamous cell carcinoma (ESCC). Moreover, association of LINC02820 with MYH9 protein protects MYH9 from proteasomal degradation, thus upregulating MYH9 to promote tumor growth and metastasis in ESCC.

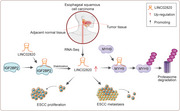

## 1 Introduction

Esophageal cancer (ESCA), a highly prevalent digestive tract malignancy, is histologically categorized into two main subtypes: esophageal adenocarcinoma and esophageal squamous cell carcinoma (ESCC) [1]. In China, the majority of ESCA cases are diagnosed at late‐stage ESCC due to the lack of early symptomatic presentation, contributing to its high mortality rate. Despite advances in treatment strategies, such as surgery and chemotherapy, the 5‐year survival rate for ESCC patients remains unsatisfactory [2, 3]. These clinical challenges underscore the urgent need to decipher the molecular mechanisms driving ESCC tumorigenesis and development.

Long noncoding RNAs (lncRNAs) are a class of RNA transcripts > 200 nucleotides generally lacking protein‐coding potential. Accumulating evidence indicates that lncRNAs perform important regulatory functions at transcriptional, posttranscriptional, and translational levels under physiological conditions [4, 5]. Their abnormal expression can disrupt key biological processes and contribute to disease pathogenesis, including cancer [6, 7, 8]. For examples, the ultra‐conserved lncRNA THOR exhibits an elevated expression in multiple cancers and promotes tumor growth by enhancing cell proliferation [9] and the lncRNA H19 shows a remarkably increased expression in bladder cancer and is implicated in metastasis through modulating epithelial–mesenchymal transition [10]. Recent studies have revealed widespread dysregulation of lncRNA expression in ESCC [11]. To date, only a limited number of lncRNAs have been functionally characterized, exhibiting diverse regulatory roles in ESCC progression. For instance, super‐enhancer‐driven overexpression of LINC01503 promotes ESCC cell proliferation, migration, and invasion via activating ERK signaling [12]. Both LINC00680 and LINC00022 can enhance tumor growth, correlating with poor prognosis [13, 14]. Additionally, lncRNAs such as SLC25A21‐AS1 and NORAD regulate critical oncogenic pathways, including the NPM1/c‐Myc and miR‐224‐3p/MTDH axes, thereby modulating tumor aggressiveness and chemoresistance in ESCC [15, 16]. Besides, the upregulation of the lncRNA AGPG activates glycolytic flux and accelerates cell cycle progression in ESCC [17]. Despite these advances, the biological roles of most lncRNAs in ESCC remain unexplored, warranting further investigations.

LncRNAs regulate gene expression through interactions with DNA, RNA, or RNA‐binding protein (RBP), thereby influencing key hallmarks of caner [4, 18]. For examples, the lncRNA MEG3 regulates the TGF‐β pathway genes through formation of RNA–DNA triplex structures [19], while H19 acts as a molecular sponge for miR‐675 to regulate cell proliferation [20]. LncRNA–protein interaction is a more commonly observed mechanism, which modulates protein function or regulates protein–protein interaction. For instance, the p53‐induced lncRNA–p21 mediates global gene repression during the p53 response by its physical association with hnRNP K [21]. Similarly, the lncRNA BREA2 stabilizes NICD1 by impairing the complex formation of NICD1 protein and the E3 ligase WWP2, leading to Notch signaling activation and lung metastasis [22]. On the other hand, lncRNA–RBP complex may affect lncRNA abundance. In gallbladder cancer, the RBP HuR stabilizes the lncRNA–HGBC, enhancing its oncogenic function and promoting metastasis [23]. Therefore, understanding how lncRNA–protein complex regulate ESCC development may facilitate the identification of novel therapeutic targets.

In this study, we identified significant upregulation of LINC02820 in ESCC tumors. Mechanistically, we found that insulin‐like growth factor 2 mRNA‐binding protein 2 (IGF2BP2) binds to and stabilizes LINC02820, leading to its elevated expression in ESCC cells. Functional assays employing both gain‐ and loss‐of‐function approaches revealed that LINC02820 promotes tumor cell proliferation and migration in vitro and enhances tumor growth and metastasis in vivo. Further mechanistic investigation demonstrated that LINC02820 interacts with MYH9 protein and protects it from ubiquitination–proteasomal degradation. Therefore, our findings identify the oncogenic functions of LINC02820 in ESCC progression and underscore its potential as a therapeutic target.

## 2 Results

### 2.1 LINC02820 Expression is Upregulated in ESCC

**FIGURE 1:**
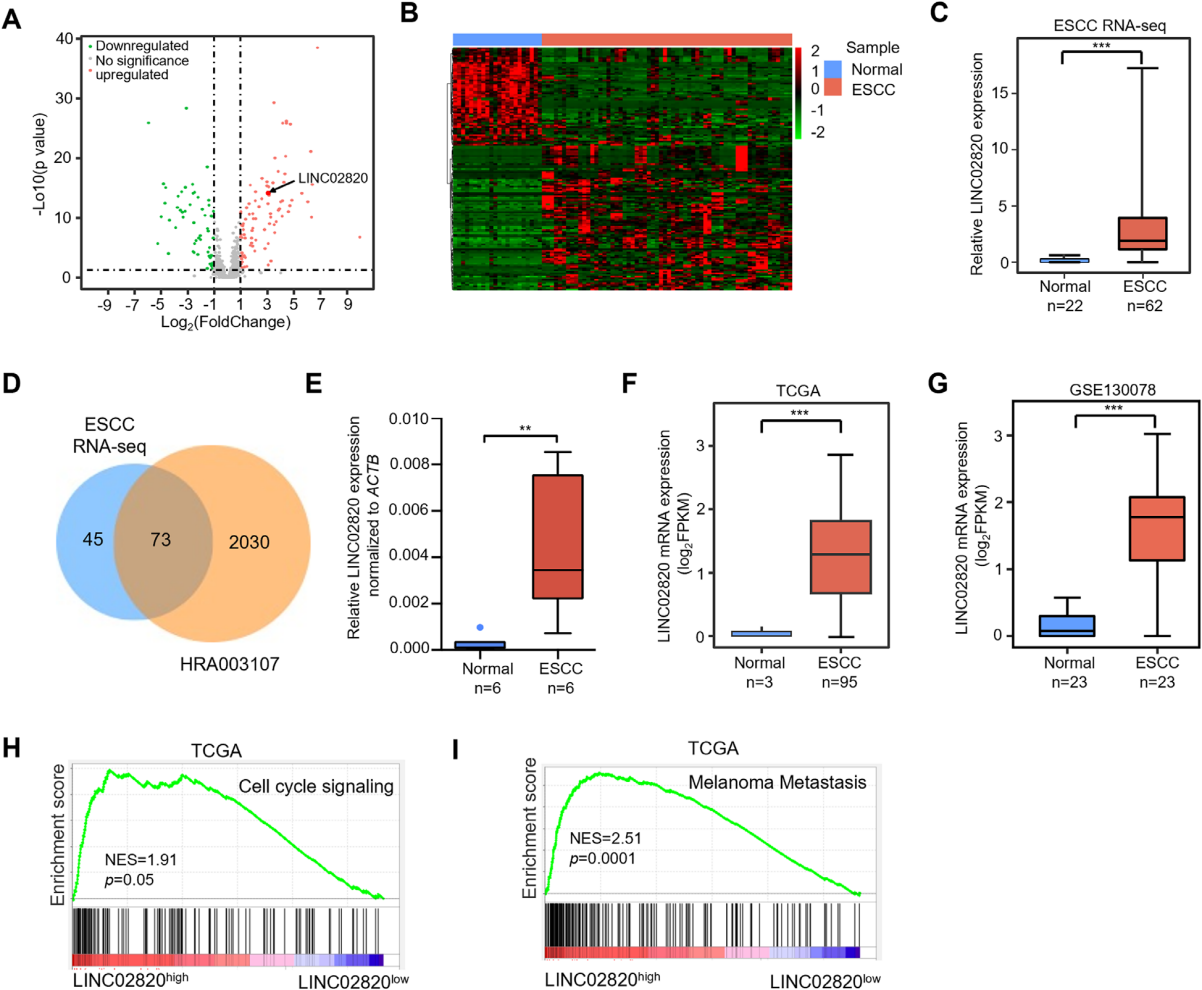
LINC02820 expression is upregulated in ESCC. (A) Volcano plot and (B) hierarchical cluster plot exhibiting differentially expressed lncRNAs between ESCC tumors and paired corresponding normal tissues from our Ribo‐Zero RNA‐seq data. (C) LINC02820 expression in ESCC tumors and paired normal tissues from our RNA‐Seq data. (D) Venn diagrams showing the overlap of differentially expressed lncRNAs (gene‐level) between our ESCC RNA‐seq data (118 genes from 151 transcripts) and the HRA003107 dataset (2103 genes). (E) RT‐qPCR analysis of LINC02820 expression in ESCC tumors and adjacent normal tissues from 6 patients. LINC02820 levels were normalized to *ACTB* mRNAs. (F and G) LINC02820 expression between ESCC tumors and normal tissues in TCGA–ESCC (F) and GSE130078 (G) datasets. (H and I) Gene Set Enrichment Analysis showing the association of LINC02820 abundance with cell cycle (H) and metastasis (I) pathways in the TCGA dataset.

To identify differentially expressed lncRNAs in ESCC, we performed Ribo‐Zero high‐throughput RNA‐Sequencing followed by comprehensive bioinformatics analysis. The results revealed 91 upregulated and 60 downregulated lncRNAs in ESCC tumor tissues compared with normal controls (Figure 1A,B and Table S1). Notably, LINC02820 exhibited an 8.5‐fold increase in ESCC tumors, while C5orf66‐AS1 and AC004816.1 showed significant downregulation (Figures 1C and S1A). To verify our RNA‐Sequencing results, we compared our data with another ESCC cohort of HRA003107 data and observed that approximately 62% of differentially expressed lncRNAs we identified overlapped with the HRA003107 data (Figure 1D). Next, we collected independent set of paired ESCC samples to measure lncRNA levels by real‐time quantitative polymerase chain reaction (RT‐qPCR), and the results showed that most of tested lncRNAs exhibited consistent expression patterns with our sequencing data (Figures 1E and S1B). Moreover, bioinformatics analyses from The Cancer Genome Atlas (TCGA) and GSE130078 datasets confirmed that LINC02820 was dramatically upregulated in ESCC tissues (Figure 1F,G), suggesting its potential oncogenic role. Subsequently, we performed gene set enrichment analysis (GSEA) to explore biological processes and signaling pathways regulated by LINC02820. The results showed that higher LINC02820 expression is significantly enriched in cell cycle regulation and metastasis‐related pathways in ESCA (Figure 1H,I), suggesting its functional involvement in ESCA cell proliferation and migration.

### 2.2 Characterization and Subcellular Distribution of LINC02820 in ESCC Cells

**FIGURE 2:**
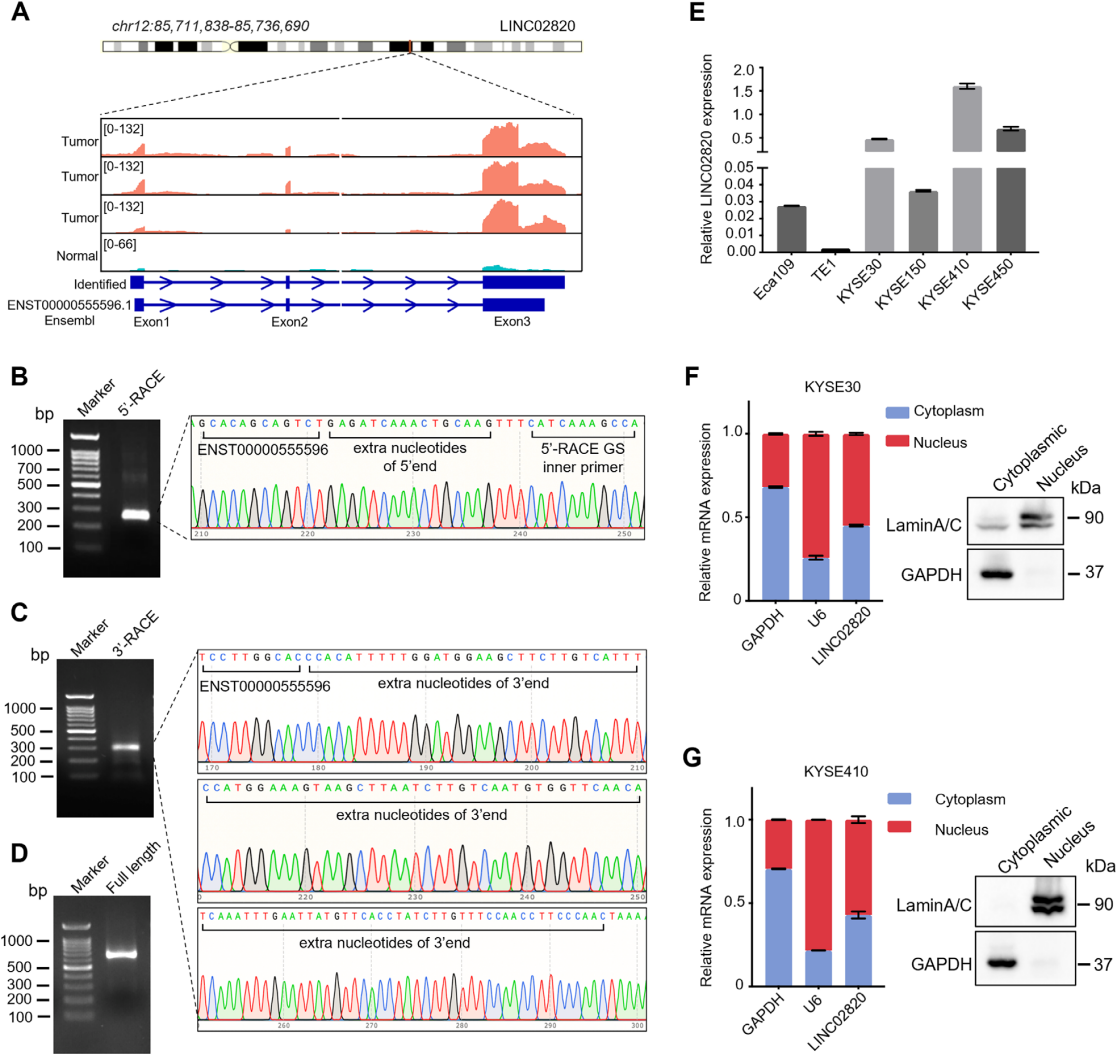
Characterization and subcellular distribution of LINC02820 in ESCC cells. (A) Identification of LINC02820 variant in ESCC tumors. Upper panel, the genomic loci of LINC02820; middle panel, our high‐throughput RNA‐Seq profiles in LINC02820 loci; lower panel, schematic diagram of ENST00000555596. (B and C) Agarose gel electrophoresis of PCR products generated by 5′‐RACE (B) and 3′‐RACE (C), Sanger sequencing confirms extra nucleotides of LINC02820 variant. (D) Agarose gel electrophoresis of the full‐length LINC02820 variant generated by RT‐PCR. (E) RT‐qPCR measurement of LINC02820 levels in ESCC cells. LINC02820 expression is normalized to *ACTB* mRNAs. (F and G) Subcellular distribution of LINC02820 in KYSE30 (F) and KYSE410 (G) cells determined by RT‐qPCR assay. Western blot assays with GAPDH and Lamin A/C antibodies confirm effective cytoplasmic/nuclear fractionation. *GAPDH* mRNA and *U6* RNA transcripts are used as the cytoplasmic and nucleus indicators, respectively.

Analysis of our high‐throughput RNA‐Seq data revealed that LINC02820 consists of three exons and exhibited extended read lengths compared with the reference sequence (ENST00000555596) in the Ensembl database (Figure 2A). To experimentally validate the transcription initiation and termination sites of LINC02820, we performed 5′ and 3′ rapid amplification of cDNA ends (RACE), followed by Sanger sequencing. Both RACE assays yielded single distinct bands, with sequencing results identifying 16 additional nucleotides at the 5′ end and 118 extra nucleotides at the 3′ end that were absent in the database annotation (Figure 2B,C). Subsequent amplification and sequencing of the full‐length transcript confirmed LINC02820 as a 653‐nucleotide RNA (Figures 2D and S2). Consistent with its classification as a lncRNA, bioinformatics analysis using the Coding Potential Calculator (http://cpc.cbi. pku.edu.cn/) confirmed that LINC02820 lacks protein‐encoding capacity.

To investigate the functional role of LINC02820 in ESCC, we first examined its expression patterns across a panel of human ESCC cell lines. As shown in Figure 2E, LINC02820 were highly expressed in KYSE30, KYSE410, and KYSE450 cells, while it exhibited lower expression in Eca109 and TE1 cells.

To determine the subcellular localization of LINC02820, we prepared cytoplasmic and nuclear fractions of KYSE410 and KYSE30 cells to measure LINC02820 levels. Western blot analysis confirmed effective separation of cytoplasmic and nuclear fractions. Subsequent RT‐qPCR analysis showed that LINC02820 is distributed in both cellular compartments (Figure 2F,G), implying its potential involvement in both transcriptional and posttranscriptional regulatory mechanisms during ESCC progression.

### 2.3 LINC02820 Promotes ESCC Tumor Growth and Metastasis

**FIGURE 3:**
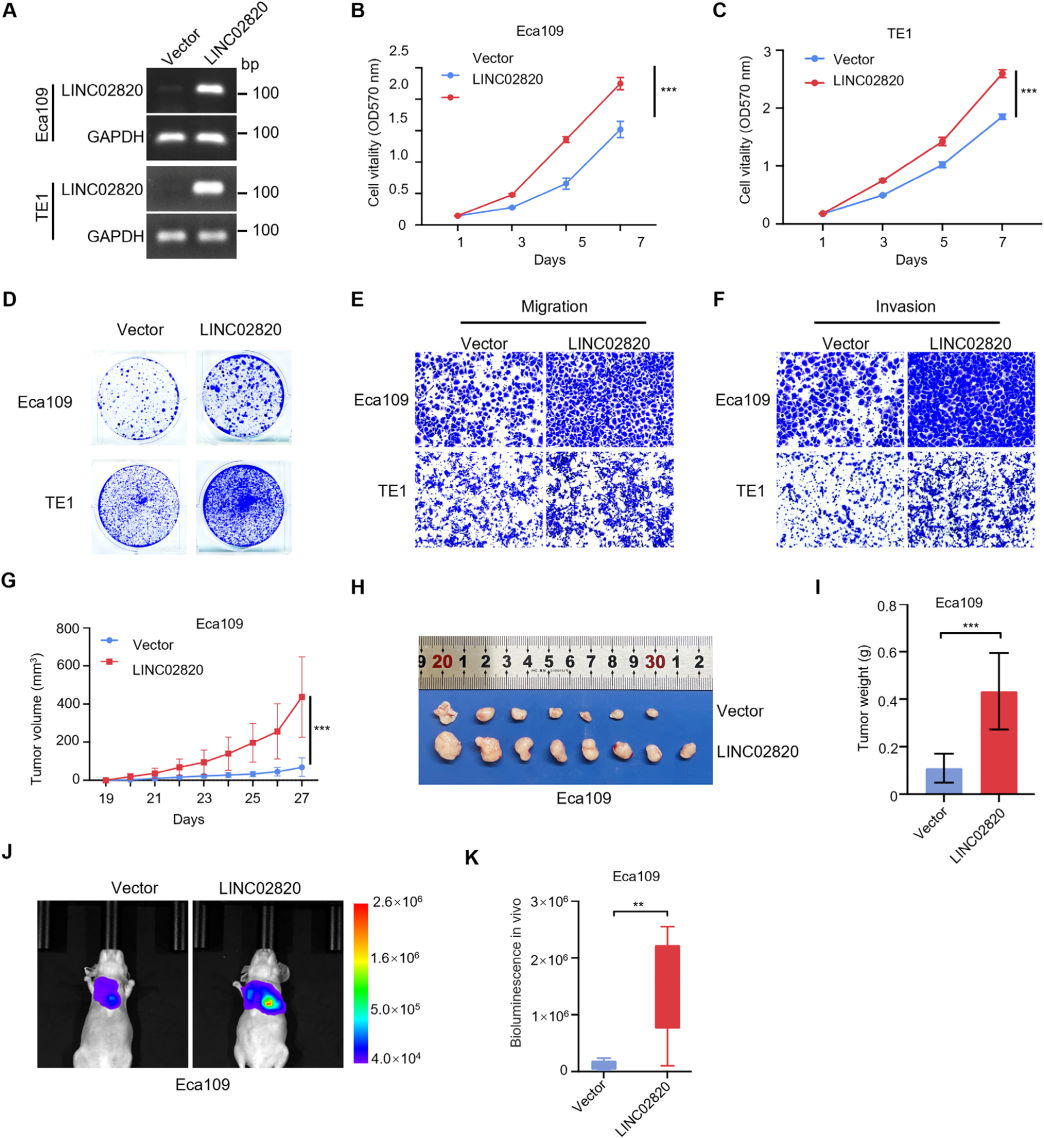
LINC02820 promotes tumor growth and metastasis. (A) Semi‐quantitative RT‐PCR and agarose gel electrophoresis show successful LINC02820 overexpression in Eca109 and TE1 cells. (B and C) MTT assays indicate that LINC02820 overexpression promotes cell growth in Eca109 (B) and TE1 (C) cells. Data are shown as mean ± s.d. *n* = 4. (D) Colony formation assays of ESCC cells with or without LINC02820 overexpression. (E and F) Representative Transwell migration (E) and invasion (F) images of ESCC cells with or without LINC02820 overexpression. (G–I) LINC02820 overexpression enhances ESCC tumor growth (*n* = 8/group). Tumors (H) and their weights (I) are displayed at the end point. (J and K) Representative bioluminescence images (J) of nude mice after tail‐vein injection with Eca109 stable cells with or without LINC02820 overexpression (*n* = 7/group), and their bioluminescence intensities in the lungs are quantified (K).

Given the relatively low endogenous expression of LINC02820 in Eca109 and TE1 cells (Figure 2E), we selected these cells to successfully establish stable LINC02820‐overexpressing cells via lentiviral transduction, as evidenced by RT‐PCR analyses (Figure 3A). Functional studies showed that ectopically expressed LINC02820 significantly enhanced cell proliferative capacity in both Eca109 and TE1 cells, as demonstrated by both MTT assays (Figure 3B,C) and colony formation experiments (Figure 3D). Furthermore, Transwell assays indicated that LINC02820 overexpression markedly promoted cell migration and invasion in both cell lines (Figure 3E,F). Together, these results demonstrate that LINC02820 enhances cell proliferation, migration, and invasion abilities of ESCC cells.

To evaluate the effects of LINC02820 in vivo, we performed subcutaneous xenograft assays using LINC02820‐overexpressing Eca109 cells in BALB/c nude mice. Tumors derived from LINC02820‐overexpressing cells exhibited significantly increased growth rates (Figure 3G,H) and final tumor weights (Figure 3I) compared with control groups. Furthermore, tail‐vein injection experiments indicated that LINC02820 overexpression substantially enhanced the metastatic potential of ESCC cells, resulting in significantly more lung metastasis (Figure 3J,K). These in vivo data collectively demonstrate that LINC02820 promotes both tumor growth and distant metastasis, supporting its oncogenic role in ESCC progression.

### 2.4 Targeting LINC02820 Expression Inhibits ESCC Tumor Growth and Metastasis

**FIGURE 4:**
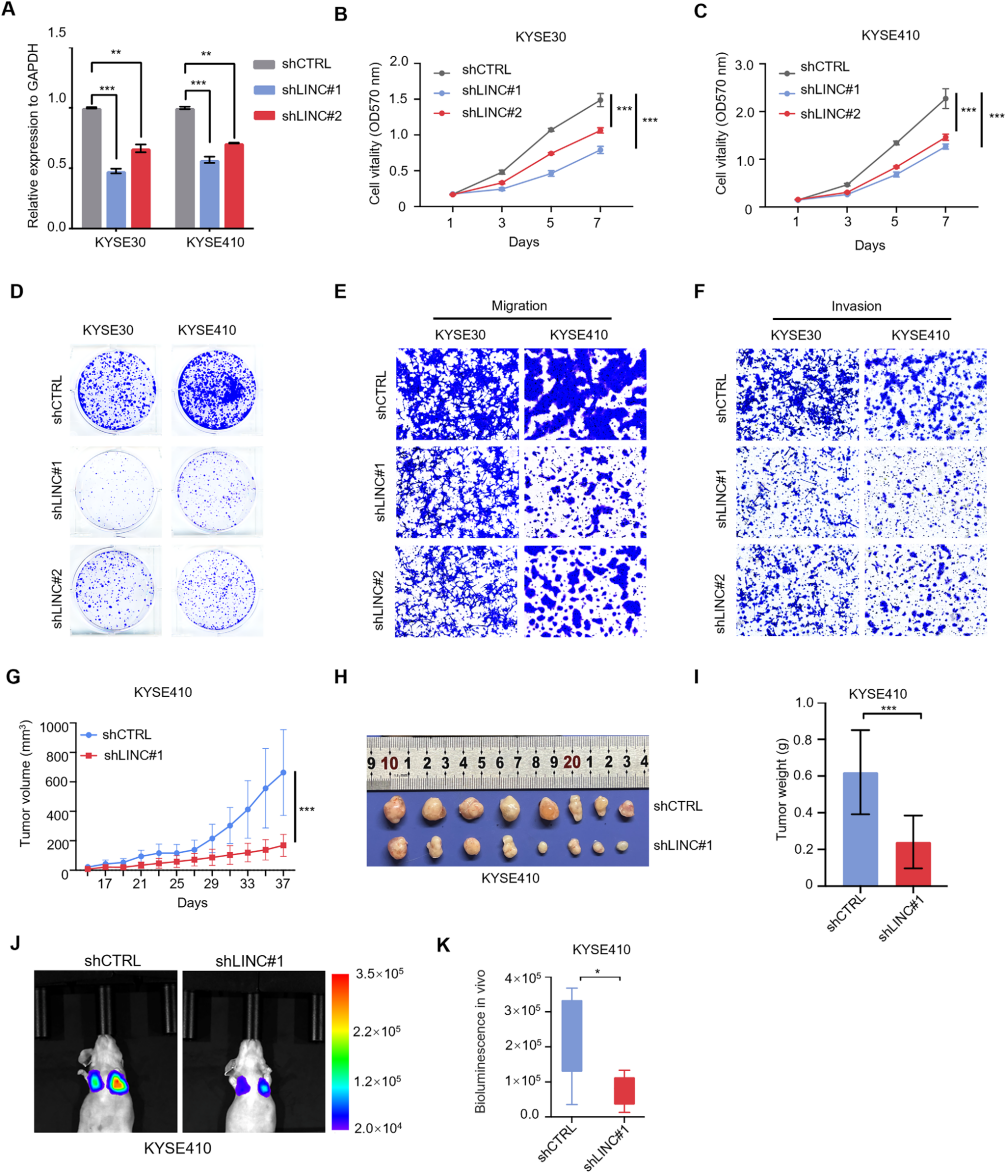
Targeting LINC0282 expression inhibits ESCC tumor growth and metastasis. (A) RT‐qPCR assays showing efficient LINC02820 knockdown by shNRAs in ESCC cells. LINC02820 expression is normalized to *GAPDH* mRNAs. (B, C) MTT assays show that LINC02820 depletion inhibits cell growth in KYSE30 (B) and KYSE410 cells (C). Data are shown as mean ± s.d. *n* = 4. (D) Colony formation assays of ESCC cells with or without LINC02820 knockdown. (E and F) Representative Transwell migration (E) and invasion (F) images of ESCC cells with or without LINC02820 knockdown. (G–I) LINC02820 depletion represses ESCC tumor growth (*n* = 8/group). Tumors (H) and their weights (I) are displayed at the end point. (J and K) Representative bioluminescence images of nude mice after tail‐vein injection with KYSE410 stable cells with or without LINC02820 knockdown (*n* = 7/group), and their bioluminescence intensities in the lungs are quantified (K).

To further validate the biological function of LINC02820 in ESCC progression, we generated stable knockdown cell lines using two independent short‐hairpin RNAs targeting LINC02820 in KYSE30 and KYSE410 cells. Quantitative RT‐PCR analysis confirmed shRNAs can efficiently knock down LINC02820 expression in both cell lines (Figure 4A). Next, MTT assays showed that LINC02820 depletion significantly inhibited cell proliferation (Figure 4B,C), which was further validated by colony formation experiments (Figure 4D). Furthermore, Transwell assays demonstrated that LINC02820 silencing markedly reduced cell migration and invasion ability (Figure 4E,F).

To validate the tumor‐promoting function of LINC02820 in vivo, we established xenograft models using LINC02820‐knockdown KYSE410 cells. Compared with control (shCTRL) groups, LINC02820 depletion (shLINC) significantly inhibited tumor growth (Figure 4G,H) and reduced final tumor weights (Figure 4I). Moreover, tail‐vein injection experiments of KYSE410 stable cells indicated that LINC02820 silencing substantially diminished lung metastatic colonization (Figure 4J,K). Together with our overexpression results (Figure 3), these gain‐ and loss‐of‐function studies support the essential role of LINC02820 in promoting ESCC tumor growth and metastasis.

### 2.5 IGF2BP2 Enhances LINC02820 Stability in ESCC Cells

**FIGURE 5:**
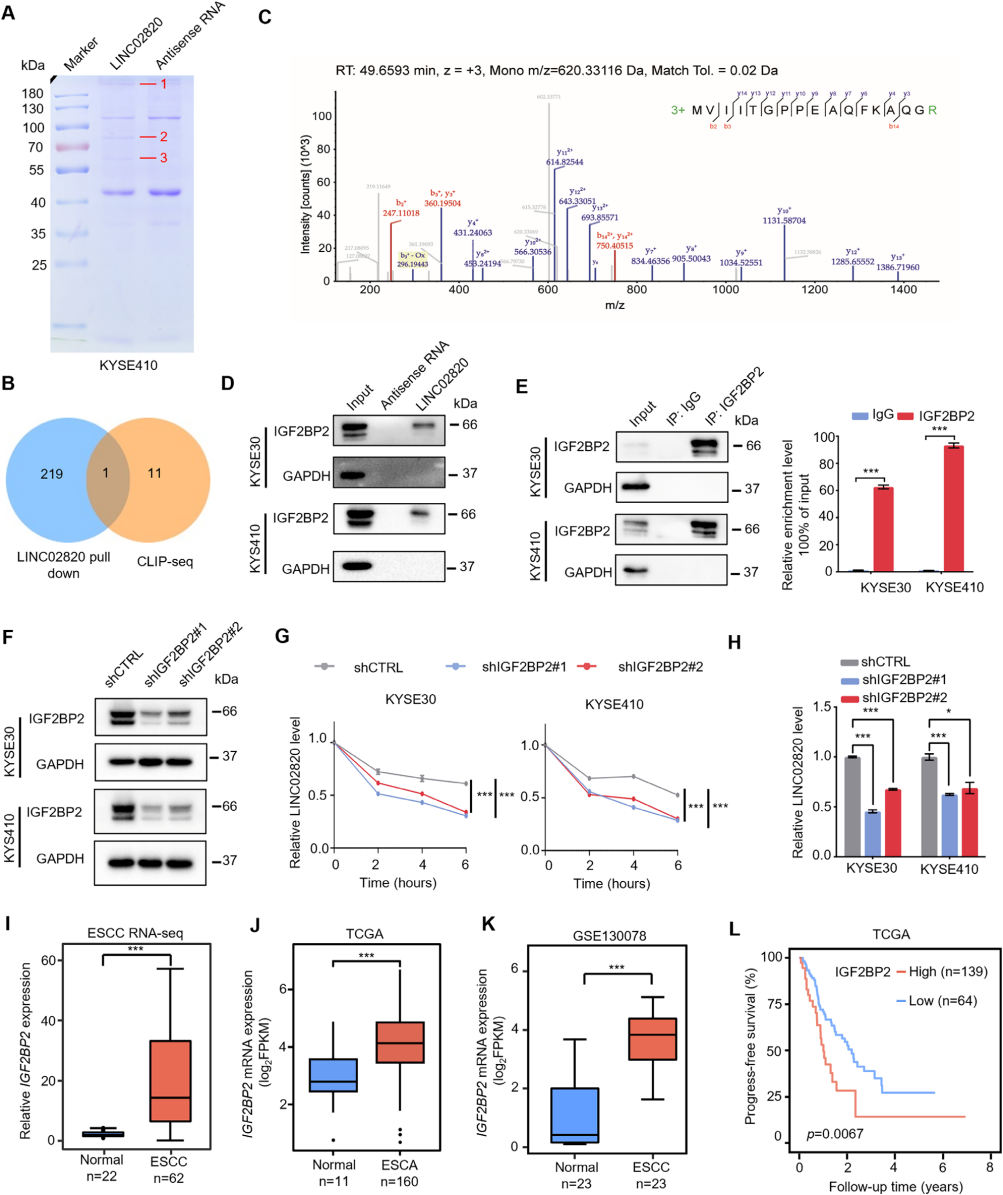
IGF2BP2 enhances LINC02820 stability in ESCC cells. (A) Coomassie blue staining of LINC02820‐interacting proteins pulled down by the biotinylated LINC02820 or antisense transcripts from KYSE410 cell extracts. Proteins indicated by red numbers were subjected to mass spectrometry analysis. (B) Venn diagrams showing the overlap of LINC02820‐associated proteins between our mass spectrometry data and CLIP‐seq dataset. (C) A representative IGF2BP2 peptide identified by LC–MS/MS. (D) Immunoblot assays show that IGF2BP2 was specifically pulled down by the biotinylated LINC02820 from KYSE30 or KYSE410 cell extracts. (E) RIP assays with anti‐IGF2BP2 antibody indicate the association of LINC02820 with IGF2BP2 in KYSE30 or KYSE410 cells. (F) Immunoblot assays show efficient IGF2BP2 knockdown by shRNAs in KYSE30 and KYSE410 cells. (G) RT‐qPCR analyses of LINC02820 levels in ESCC cells treated with actinomycin D for indicated times. (H) RT‐qPCR analyses show steady‐state levels of LINC02820 in control and IGF2BP2‐depletion ESCC cells. LINC02820 expression is normalized to *GAPDH* mRNA. (I) *IGF2BP2* mRNA levels between ESCC tumors and paired normal tissues in our RNA‐Seq data. (J and K) *IGF2BP2* mRNA expression between tumors and normal tissues in TCGA–ESCA (J) and GSE130078 (K) datasets. (L) Kaplan–Meier survival curves of individuals with *IGF2BP2* mRNA expression in the TCGA dataset.

Accumulating evidence demonstrates that lncRNAs functionally interact with RBPs to regulate gene expression or to be regulated by proteins. To identify LINC02820‐associating proteins in ESCC, we performed RNA pull‐down assays using in vitro transcribed sense or antisense LINC02820, Coomassie blue staining revealed several proteins (>180, >70, and >55 kDa) specifically enriched by biotinylated LINC02820 from both KYSE410 (Figure 5A) and KYSE30 cell lysates (Figure S3A), which were subsequently excised for mass spectrometry analyses (Tables S2–S4). Moreover, we analyzed CLIP‐seq data from starBase v2.0 and identified 12 potential LINC02820‐interacting RBPs (Table S5). Notably, IGF2BP2 was identified by both our mass spectrometry data and CLIP‐seq analyses (Figure 5B,C), supporting it as a binding partner of LINC02820. Next, we validated this interaction through independent RNA pull‐down assays followed by immunoblotting, which confirmed specific association between IGF2BP2 and LINC02820 (Figure 5D). Furthermore, reciprocal RBP immunoprecipitation using anti‐IGF2BP2 antibody also revealed significant enrichment of LINC02820 in the immunoprecipitated fraction compared with controls (Figure 5E). Together, these results demonstrate that IGF2BP2 binds to LINC02820 in ESCC cells.

Given the established role of IGF2BP2 protein in stabilizing target RNAs, such as *Wnt7B* mRNA and the lncRNA DANCR [24, 25, 26, 27], we wonder if IGF2BP2 similarly regulates LINC02820 stability. To this end, we generated stable IGF2BP2‐depletion cell lines using two different shRNAs in both KYSE30 and KYSE410 cells. First, the immunoblotting results confirmed that IGF2BP2 were efficiently knocked down in both cell lines (Figure 5F). To assess RNA stability, we treated cells with actinomycin D to block new RNA synthesis and monitored LINC02820 levels over time by RT‐qPCR. As shown in Figure 5G, LINC02820 exhibited significantly accelerated decay following IGF2BP2 depletion in both cell lines, indicating that IGF2BP2 enhances LINC02820 stability. Consistent with this finding, IGF2BP2 knockdown substantially reduced steady‐state LINC02820 expression levels (Figure 5H).

To explore the clinical relevance of LINC02820 and IGF2BP2 in ESCC, we first analyzed their expression patterns in our sequencing dataset and found that IGF2BP2 expression were significantly elevated in ESCC tumors compared with paired adjacent normal tissues (Figure 5I), which was further validated in both TCGA (Figure 5J) and GSE130078 datasets (Figure 5K). Notably, we observed a strong positive correlation between IGF2BP2 and LINC02820 expression levels (Figure S3B), suggesting that IGF2BP2‐mediated regulation may contribute to LINC02820 upregulation in ESCA. Importantly, higher IGF2BP2 expression was significantly associated with worse clinical outcomes in ESCA patients (Figure 5L), underscoring its potential prognostic value.

Considering that the lncRNA BREA2 has been shown to interact with and stabilize NICD1 protein [22], we sought to determine whether LINC02820 similarly regulates IGF2BP2 expression. However, neither LINC02820 knockdown nor overexpression significantly altered IGF2BP2 protein levels (Figure S3C,D). These results indicate that while IGF2BP2 stabilizes LINC02820, this interaction does not reciprocally regulate IGF2BP2 expression in ESCC cells.

### 2.6 LINC02820 Interacts with and Increases MYH9 Protein in ESCC Cells

**FIGURE 6:**
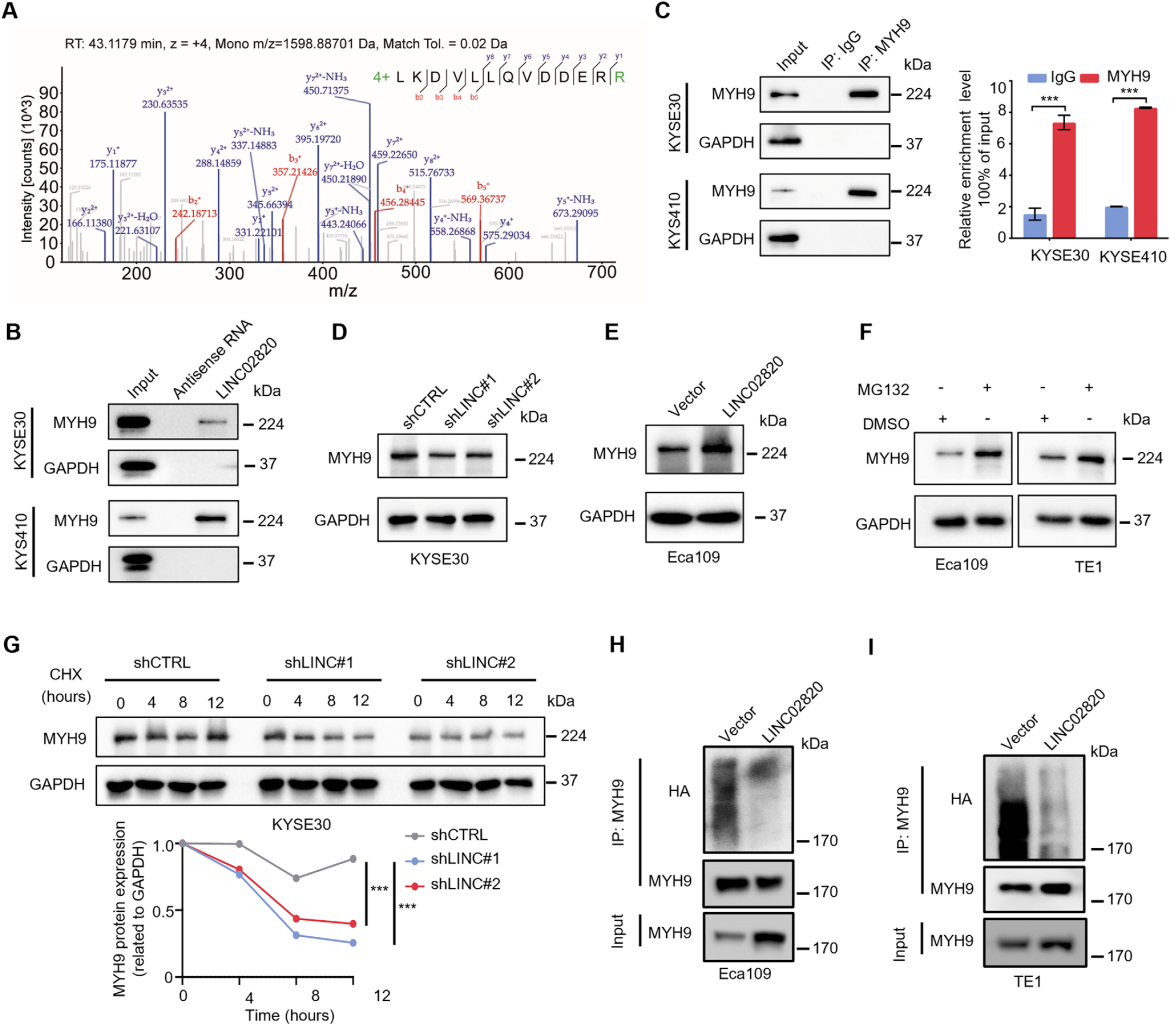
LINC02820 interacts with and increases MYH9 protein in ESCC cells. (A) A representative MYH9 peptide identified by LC–MS/MS. (B) Pulldown of MYH9 by the biotinylated LINC02820 from KYSE30 or KYSE410 cell extracts. (C) RIP assays using anti‐MYH9 antibody indicate the association of LINC02820 with MYH9 protein in KYSE30 and KYSE410 cells. (D and E) Effects of LINC02820 knockdown (D) and overexpression (E) on MYH9 expression in ESCC cells. (F) Effects of MG132 treatment on MYH9 protein levels in ESCC cells. (G) Immunoblot analyses of MYH9 levels in different KYSE30 cells treated with cycloheximide for indicated times. (H and I) Polyubiquitination status of MYH9 proteins immunoprecipitated from Eca109 (H) and TE1 cells (I) with or without LINC02820 overexpression.

Our mass spectrometry analysis identified Myosin‐9 (MYH9) as a high‐confidence LINC02820‐interacting protein, supported by robust identification metrics including a high mass spectrometry score (Sum PEP Score = 821.942), numerous unique peptides (127), and extensive sequence coverage (50%) (Figure 6A and Table S2). Given MYH9's established roles in maintaining cell morphology, cell adhesion and cytokinesis [28], and its association with postoperative ESCC recurrence [29], we performed RNA pull‐down experiments and immunoblotting assays to confirm the interaction between LINC02820 and MYH9. The results showed that MYH9 protein was specifically captured from ESCC cell lysates by LINC02820, but not by antisense RNA (Figure 6B). Furthermore, RIP assays confirmed endogenous association between LINC02820 and MYH9 in both KYSE30 and KYSE410 cells (Figure 6C). Increasing studies report that lncRNAs can modulate protein stability [30, 31], so we wonder if LINC02820 has an effect on MYH9 protein abundance. The results showed that LINC02820 depletion decreased MYH9 protein levels (Figures 6D and S4C), whereas LINC02820 overexpression increased MYH9 protein abundance (Figures 6E and S4D). Notably, neither LINC02820 knockdown or overexpression had impact on MYH9 mRNA levels (Figure S4A,B), implying that LINC20280 regulates MYH9 expression through posttranslational mechanisms rather other transcriptional control.

### 2.7 LINC02820 Protects MYH9 from Ubiquitination‐Mediated Proteasomal Degradation

Since MYH9 is known to be regulated by ubiquitin–proteasome pathway [32, 33], we first examined MYH9 protein levels after treating cells with the proteasome inhibitor MG132. As shown in Figure 6F, MG132 treatment effectively increased MYH9 abundance in both Eca109 and TE1 cells. To directly assess MYH9 stability, we treated cells with cycloheximide (CHX) to block protein synthesis for indicated times, and then measured MYH9 protein by immunoblotting analysis. The results showed that MYH9 protein levels declined more quickly after LINC02820 knockdown in ESCC cells (Figures 6G and S4E), indicating that LINC02820 prolongs the half‐life of MYH9 protein. Given the critical role of polyubiquitination in protein degradation [34], we next evaluated the effects of LINC02820 on MYH9 ubiquitination status. The result showed that LINC02820 overexpression significantly decreased polyubiquitination levels of MYH9 (Figure 6H,I). Taken together, our results demonstrate that LINC02820 interacts with MYH9 and protects it from ubiquitination‐mediated proteasomal degradation.

## 3 Discussion

ESCC is a highly aggressive malignancy with poor clinical outcomes, underscoring the need to elucidate its molecular mechanisms and identify novel therapeutic targets. Recently, some lncRNAs, such as TMEM44‐AS1 and LINK‐A, have been reported to participate in ESCC tumor growth and metastasis [35, 36], indicating the important role of lncRNAs in ESCC progression. However, given the vast repertoire of lncRNAs expressed in cells, the functional characterization of dysregulated lncRNAs in ESCC remains largely unexplored. In this study, we employed high‐throughput RNA‐sequencing and bioinformatics analysis to identify LINC02820 as one of the most significantly upregulated lncRNAs in ESCC tumors compared with normal tissues (Figure 1A–C), which was further validated by RT‐qPCR assays (Figure 1E), TCGA dataset (Figure 1F), and GSE130078 dataset analysis (Figure 1G). These results strongly suggest the potential oncogenic role of LINC02820 in ESCC progression.

RNA abundance is tightly regulated through both transcriptional and posttranscriptional mechanisms. For example, the transcription factor MYC activates LINC00346 expression by binding to promoter regions, and elevated LINC00346 expression promotes gastric cancer progression [37]. Posttranscriptionally, RBPs may modulate RNA stability through sequence‐specific interactions. For instance, HuR stabilizes target mRNAs (including c‐MYC and p21) by recognizing AU‐rich elements (AREs) in their 3′ untranslated regions (3′ UTRs) [38]. Our study identified the association of IGF2BP2 with LINC02820, as evidenced by RNA pull‐down assays (Figure 5D) and RBP immunoprecipitation (Figure 5E). Moreover, IGF2BP2 depletion shorten the half‐life of LINC02820 (Figure 5G) and thus decreased steady‐state LINC02820 levels (Figure 5H). Consistent with the previous report [39], we found IGF2BP2 overexpression in ESCC, which may contribute to elevated LINC02820 expression in tumors. While IGF2BP family proteins are reported to recognize “CAUC” or “UGGAC” motifs [40], the specific binding sequence within LINC02820 requires further characterization.

Although LINC02820 was found to be dysregulated in ESCC [41], its accurate sequence has not been validated. In this study, we employed RACE technologies and Sanger sequencing to experimentally identify the transcription initiation and termination sites of LINC02820 (Figure 2B,C). Moreover, the full‐length of LINC02820 was successfully amplified for sequencing (Figure 2D). Our analysis reveals that LINC02820 is located at the 12q21.31 region. Compared with the reference sequence (ENST00000555596 in Ensembl database; NR_183553 in NCBI database), LINC02820 we identified is 695 nucleotides in length, with 16 additional nucleotides at the 5′‐end and 118 additional nucleotides at the 3′‐end (Figure S2), indicating that LINC02820 we identified is a novel transcript variant in ESCC.

To investigate the functional role of LINC02820 in ESCC, we established stable cell lines overexpressing this lncRNA. Functional assays indicated that LINC02820 significantly enhanced both proliferative and migratory capacities of ESCC cells (Figure 3A–F). Conversely, LINC02820 depletion using two independent shRNAs effectively suppressed cell proliferation and migration (Figure 4), confirming its oncogenic functions in ESCC cells. These in vitro findings were further corroborated by in vivo studies showing that LINC02820 promoted tumor growth and metastasis in xenograft models (Figure 3G–K). Interestingly, Wang et al. [41] reported that LINC02820 primarily promotes cell migration without impacting proliferation. This functional discrepancy may stem from differences between the transcript variant we identified (containing additional 5′ and 3′ sequences) and previously reported isoforms, suggesting that distinct LINC02820 transcripts may adopt different conformations to exert specific biological functions.

Compelling evidence indicates that lncRNAs modulate gene expression through protein interactions at posttranscriptional level. For example, the p53‐responsive lncRNA GUARDIN acts as a scaffold to promote BRCA1–BARD1 heterodimer formation, thereby regulating BRCA1 protein level and genomic stability [30]. Similarly, in papillary thyroid carcinoma, the lncRNA AB074169 interacts with KHSRP protein to downregulate its expression and inhibit cell proliferation [42]. In this study, we identified MYH9 as a LINC02820‐interacting protein through RNA pull‐down and mass spectrometry (Figure 6A), with subsequent validation by immunoblotting assays (Figure 6B) and coimmunoprecipitation experiments (Figure 6C). Through gain‐ and loss‐of‐function strategies, LINC02820 was proved to positively regulate MYH9 protein abundance (Figure 6D,E). Moreover, LINC02820 prevents MYH9 from ubiquitination‐mediated proteasomal degradation (Figure 6H,I). However, the underlying molecular mechanisms remain unclear. For instance, which E3 ubiquitin ligase mediates MYH9 degradation in this context? Are additional RBPs involved in the LINC02820–MYH9 interaction? MYH9, a cytoskeletal protein critical for cell motility and morphology, exhibits oncogenic properties in various malignancies including liver cancer, colorectal cancer, and prostate cancer [43, 44, 45]. Recent work by Li et al. [29] reported that SAMD9 promotes ESCC progression through upregulating MYH9 expression and subsequent activation of GSK3β/β‐catenin signaling. Our findings reveal an additional layer of MYH9 regulation in ESCC, highlighting the complexity of its expression control in cancer cells.

In summary, this study characterized a novel LINC02820 transcript variant that is significantly upregulated in ESCC. We demonstrated that IGF2BP2 binds to and stabilizes LINC02820, leading to its increased expression in ESCC cells. Through comprehensive functional studies, we proved that LINC02820 promotes cell proliferation and migration *in* *vitro* and accelerates tumor growth and metastasis in vivo. Mechanistically, LINC02820 interacts with MYH9 and protects it from ubiquitination‐mediated proteasomal degradation. Therefore, our findings reveal the oncogenic role of LINC02820 in ESCC progression and highlight its potential as a therapeutic target.

## 4 Materials and Methods

### 4.1 Clinical Samples

This study was approved by the Ethics Committee of West China Hospital of Sichuan University and written informed consent was obtained from patients. Fresh ESCC tumors and paired adjacent normal tissues were collected from West China Hospital.

### 4.2 Cell Culture and Construction of Stable Cells

Human esophageal squamous cells (TE1, Eca109, KYSE30, KYSE150, KYSE410, and KYSE450) and human embryonic kidney HEK293T cells were cultured in DMEM medium (Gibco) supplemented with 10% fetal bovine serum and 1% penicillin/streptomycin at 37°C in a humidified 5% CO_2_ incubator. To prepare lentivirus, HEK293T cells were cotransfected with the lentiviral plasmid and packaging plasmids psPAX2 and pMD2G. At 48 h after transfection, supernatants containing lentivirus were collected to infect ESCC cells, followed by selection by puromycin to generate stable cells.

### 4.3 RNA Extraction and RT‐qPCR

Total RNAs were isolated using RNAiso Plus reagent (TaKaRa) according to the manufacturer's protocol. RNA integrity was verified prior to library preparation. After depleting ribosomal RNAs with Ribo‐Zero rRNA Removal Kit, high‐throughput RNA‐sequencing was performed by Novogene Technology Co., Ltd. (Beijing, China) to generate 150 bp paired‐end reads. For gene expression analysis, 1 µg of total RNAs were reverse transcribed using PrimeScript RT Reagent Kit (TaKaRa), followed by RT‐qPCR using TB Green Premix Ex Taq II (Tli RNase H Plus) (TaKaRa) on a QuantStudio 6 system (Life Technologies). Relative gene expression was calculated using the 2^−ΔΔCt^ method with normalization to endogenous controls. Primer sequences are listed in Table S6.

### 4.4 Transcriptome Sequencing and Bioinformatics Analysis

RNA‐seq data from 62 tumor tissues and 22 matched normal tissues of 22 ESCC patients were obtained from our previous study [46]. Raw reads were quality‐checked with FastQC (v0.11.8) and trimmed using Trimmomatic (v0.36) [47]. Clean reads were aligned to human reference genome (GRCh37.p13) using HISAT2 (v2.1.0) [48]. Transcript assembly and quantification were performed using StringTie (v2.0.4) [49], guided by the GENCODE v19 human gene annotation, and lncRNA‐specific counts and FPKM values were extracted for expression profiling.

Differential expression analysis was performed using DESeq2 (v1.32.0) [50] on lncRNAs with average FPKM ≥ 2, identifying 151 transcripts (118 genes) with fold‐change >2 and adjusted *p* value <0.05. The 118 genes were compared with 2103 dysregulated lncRNA genes from a published ESCC cohort (HRA003107, 93 pairs) [11], which used the same thresholds without expression filtering, to identify overlapping dysregulated lncRNAs.

### 4.5 RACE Experiments and Plasmid Construction

The transcription initiation and termination sites of LINC02820 were determined by FirstChoice RLM‐RACE Kit (Ambion) according to the manufacturer's instructions. The full‐length LINC02820 transcript was amplified from KYSE410 cDNAs using Phanta Super‐Fidelity DNA Polymerase (Vazyme) and subsequently cloned into pCDH–CMV–EF1–PURO for expression studies. For gene knockdown experiments, two independent shRNA oligonucleotides targeting either LINC02820 or IGF2BP2 were designed, annealed, and ligated into the vector pLKO.1‐puro at *Age*I and *Eco*RI sites. All primer and oligonucleotide sequences used in this study are listed in Table S6.

### 4.6 Cell Proliferation Assays

For colony formation assay, 3000 cells were seeded into each well of six‐well plate and cultured in complete medium for 14 days, with medium replacement every 3 days. Following incubation, colonies were fixed with 4% paraformaldehyde for 30 min at room temperature and stained with 0.5% crystal violet solution (Beyotime Biotechnology) for visualization. For MTT assays, 800 cells were seeded into each well of 96‐well plate and cultured at 37°C. At designated time points, 10 µL of MTT solution (5 mg/mL; Sigma–Aldrich) were added to each well, followed by incubation for 3 h at 37°C. The resulting formazan crystals were dissolved, and absorbance was measured at 570 nm using a BioTek microplate reader.

### 4.7 Transwell Migration and Invasion Assays

Cell migration and invasion capacities were assessed using 8 µm pore Transwell chambers (Falcon). For invasion assays, chambers were precoated with Matrigel (Corning), while migration assays used uncoated chambers. Cells were suspended in serum‐free DMEM medium containing 5% BSA and seeded into the upper chamber. The lower chamber contained DMEM medium with 10% FBS as chemoattractant. After 24‐h incubation, nonmigrated/invaded cells were removed from the upper chamber with cotton swabs. Cells on the lower membrane surface were fixed with 4% paraformaldehyde for 30 min, stained with 0.5% crystal violet solution for 30 min, and imaged using an inverted microscope.

### 4.8 Immunoblotting Assay

Proteins were extracted from cells with RIPA lysis buffer (Beyotime Biotechnology; cat#P0013B) plus Protease and Phosphatase Inhibitor Cocktails (Bimake), and protein concentrations were determined by the BCA protein assay kit (Beyotime Biotechnology) according to the manufacturer's instructions. Equal amounts of proteins were separated by 10% SDS‐PAGE and transferred to PVDF membranes (Millipore) using standard protocols. Membranes were blocked with 5% nonfat milk in TBST for 1 h at room temperature, then incubated overnight at 4°C with primary antibodies. After three washes with TBST, membranes were incubated with appropriate secondary antibodies for 1 h at room temperature. Protein bands were visualized using Enhanced Chemiluminescence Reagent (Thermo Fisher Scientific) and imaged with a ChemiDocTM XRS^+^ System (Bio‐Rad). Primary antibodies used in this study were as follows: GAPDH (CST; cat#3683S; 1:5000), Lamin A/C (Abways; cat#CY5222; 1:2000), IGF2BP2 (Proteintech; cat#11601‐1‐AP; 1:1000), MYH9 (Abcam; cat#ab55456; 1:1000), HA‐Tag (CST; cat#3724S; 1:1000).

### 4.9 Subcellular Fractionation

Cells (1 × 10^7^) were gently suspended in the hypotonic buffer (5 mM Tris–HCl, pH 7.4, 1.5 mM MgCl_2_, 150 mM NaCl, 0.5% NP‐40) and incubated on ice for 3 min to allow cell membrane disruption. Following centrifugation at 300×*g* for 2 min at 4°C, the resultant supernatant was collected as the cytoplasmic fraction. The nuclear pellet was washed twice with the hypotonic buffer and subsequently suspended in the nuclear extraction buffer. *GAPDH* mRNA and U6 RNA were used as cytoplasmic and nuclear RNAs, respectively. GAPDH and LaminA/C proteins were used as cytoplasmic and nuclear proteins, respectively.

### 4.10 in vitro Transcription and RNA Pull‐Down Assay

DNA templates for in vitro transcription were amplified by PCR and purified by DNA Gel Extraction Kit (BBI). The in vitro transcription was performed by MAXIscript T7/T3 Transcription Kit (Thermo Fisher Scientific; cat#AM1326) and Biotin RNA Labeling Mix (Roche; cat#11685597910) according to the manufacturer's instructions. For RNA pull‐down assay, 5 µg of biotinylated LINC02820 sense or antisense transcripts were denatured at 65°C for 5 min and then annealed to form appropriate secondary structures. Cells were lysed in the buffer (150 mM NaCl, 20 mM Tris–HCl, pH7.5, 0.5 mM EDTA, 0.5% Triton X‐100, protease and phosphatase inhibitors cocktail, and RNase inhibitors) using a homogenizer, followed by centrifugation at 16,000×*g* for 15 min. The resultant supernatants were incubated with sense or antisense transcripts at 4°C for 4 h, followed by adding 20 µL Streptavidin T1 (Invitrogen, cat#65601) at 4°C for another 2 h. Finally, pull‐down samples were subjected to downstream analysis. The primers used for in vitro transcription were listed in Table S6.

### 4.11 Immunoprecipitation

Cells were suspended for 20 min in the ice‐cold IP buffer (20 mM Tris–HCl, pH7.5, 150 mM NaCl, 0.5 mM EDTA, 0.5% Triton X‐100) supplemented with protease/phosphatase inhibitors and RNase inhibitors, and then lysed by homogenizer. Lysates were cleared by centrifugation at 16,000×*g* for 15 min at 4°C and incubated with indicated primary antibodies or control IgG. Immune complexes were captured using 20 µL protein A/G agarose (Millipore; cat#IP05) with rotation at 4°C for another 1 h. After washing three times with IP buffer, the immunoprecipitated proteins and RNAs were subjected to Western blot analyses and RT‐qPCR, respectively.

### 4.12 Mass Spectrometry and Data Analysis

Proteins were resolved by 10% SDS‐PAGE and stained by Coomassie brilliant blue G‐250 (Sigma). The proteins of interest were excised, destained with 40% acetonitrile/50 mM NH₄HCO₃, and then dehydrated in acetonitrile. Subsequently, samples were subjected to reduction by 25 mM NH₄HCO₃ containing 10 mM DTT and alkylation by 25 mM NH₄HCO₃ containing 40 mM iodoacetamide, followed by digestion using 5 ng/mL of trypsin in 25 mM NH₄HCO₃ at 37°C for 16 h. Then, the digestion was terminated with 0.01% formic acid. Finally, peptides were lyophilized using vacuum freeze dryer, desalted using C18 ZipTips (Millipore), and analyzed by LC–MS/MS on an Easy‐nLC 2000 HPLC system coupled to an Orbitrap ExplorisTM 480 mass spectrometer (Thermo Fisher Scientific, USA).

### 4.13 RNA and Protein Stability Assays

To measure RNA stability, cells were treated with 10 µM actinomycin D (MedChemExpress; cat#HY‐17559) to inhibit transcription. Total RNAs were extracted at indicated time points and analyzed by RT‐qPCR to determine LINC20820 half‐life. To measure protein stability, cells were treated with 50 µM CHX (Sigma–Aldrich; cat#5087390001) to block new protein synthesis. MYH9 protein levels were analyzed by Western blot at indicated time points.

### 4.14 Animal Experiments

All mouse procedures were approved by the Institutional Animal Care and Use Committee of West China Hospital, Sichuan University. Nude mice were purchased from Beijing HuaFuKang Bioscience. For xenograft model, 1 × 10^5^ cells suspended in 100 µL PBS were subcutaneously injected into 5‐week‐old male Balb/C athymic nude mice, and tumor volumes were measured and calculated using the formula (length × width^2^)/2. For lung metastasis model, 1 × 10^6^ luciferase‐labeled cells were injected into tail veins of 6‐week‐old male Balb/C athymic nude mice. Five weeks later, tumor metastasis was visualized by IVIS Spectrum system (PerkinElmer) after intraperitoneal injection of d‐luciferin. Bioluminescence intensity was quantified to reflect tumor metastasis of tumor‐bearing mice.

### 4.15 Gene Set Enrichment Analysis

GSEA was performed by the JAVA program (Version 4.2.3) with Molecular Signatures Database (MSigDB) v6.1 to investigate LINC02820‐associated pathways. The TCGA_ESCA cohort of patients were stratified into high‐ and low‐LINC02820 groups based on median expression levels. Analysis was conducted using the c2.cp.kegg_legacy.v2023.2.Hs.symbols.gmt gene set from MSigDB as the reference. Significantly enriched pathways were identified based on the highest Enrichment Score (ES) with a normalized *p* value <0.05 and a false discovery rate < 0.05.

### 4.16 Statistical Analysis

Statistical analyses were performed using GraphPad Prism software (version 9.0) to assess the differences between experimental groups. Data are presented as the mean ± standard error of the mean. Statistical significance was determined using two‐tailed Student's *t*‐tests (for comparisons between two groups). Significance thresholds were defined as follows: *p < 0.05; **p < 0.01; ***p < 0.001.

## Author Contributions

Y. P. and Y. D. L. supervised this project and revised the manuscript. X. X. and X. M. performed experiments and wrote the manuscript. W. L. and Y. Y. performed bioinformatics analysis. Y. M. contributed to animal experiments. T. Z. conducted mass spectrometric analysis. A‐Lai Gu‐Ha collected clinical samples. All authors have read and approved the final manuscript.

## Ethics Statements

This study was approved by the Institutional Animal Care and Use Committee of West China Hospital, Sichuan University (20220614004) and the Clinical Experimental and Biomedical Ethics Committee of West China Hospital, Sichuan University (2019‐537).

## Conflicts of Interest

The author Yong Peng is an editorial board member of *MedComm* but was not involved in the journal's review of or decisions related to this manuscript. All authors declared no conflict of interest.

## Supporting information



Supporting Information

Supporting Information

## Data Availability

The data included in this study are available upon request from the corresponding author.
