# Supplementary material for: The Long Noncoding RNA LINC02820 Promotes Tumor Growth and Metastasis Through Regulating MYH9 Expression in Esophageal Squamous Cell Carcinoma
Source: MedComm (2020). 2025 May 23;6(6):e70218. doi: 10.1002/mco2.70218 (PMC12099067; doi:10.1002/mco2.70218)
Supplement: Supplementary file 1 — Supporting Information [file MCO2-6-e70218-s002.pdf]

Supplementary Information for

The long non-coding RNA LINC02820 promotes tumor growth and metastasis through regulating MYH9 expression in esophageal squamous cell carcinoma

Xiaomin Xu[1#], Xinting Mao[1#], Wenrong Liu[1], Yue Ming[1], Tingting Zhang[1], Yang Yang[1], A-Lai Gu-Ha[2], Yi-Dan Lin[2*], Yong Peng[1*]

[1] Center for Molecular Oncology, Frontiers Science Center for Disease-related Molecular Network, State Key Laboratory of Biotherapy and Cancer Center, West China Hospital, Sichuan University, Chengdu, 610041, China.

[2] Department of Thoracic Surgery, West China Hospital, Sichuan University, Chengdu, 610041, China.

[#] These authors contributed equally.

[*] Corresponding authors. Email : yongpeng@scu.edu.cn (Y.P.); linyidan.academy@foxmail.com (L.Y-D.)

This PDF file includes:

Supplementary Figure 1 to 4

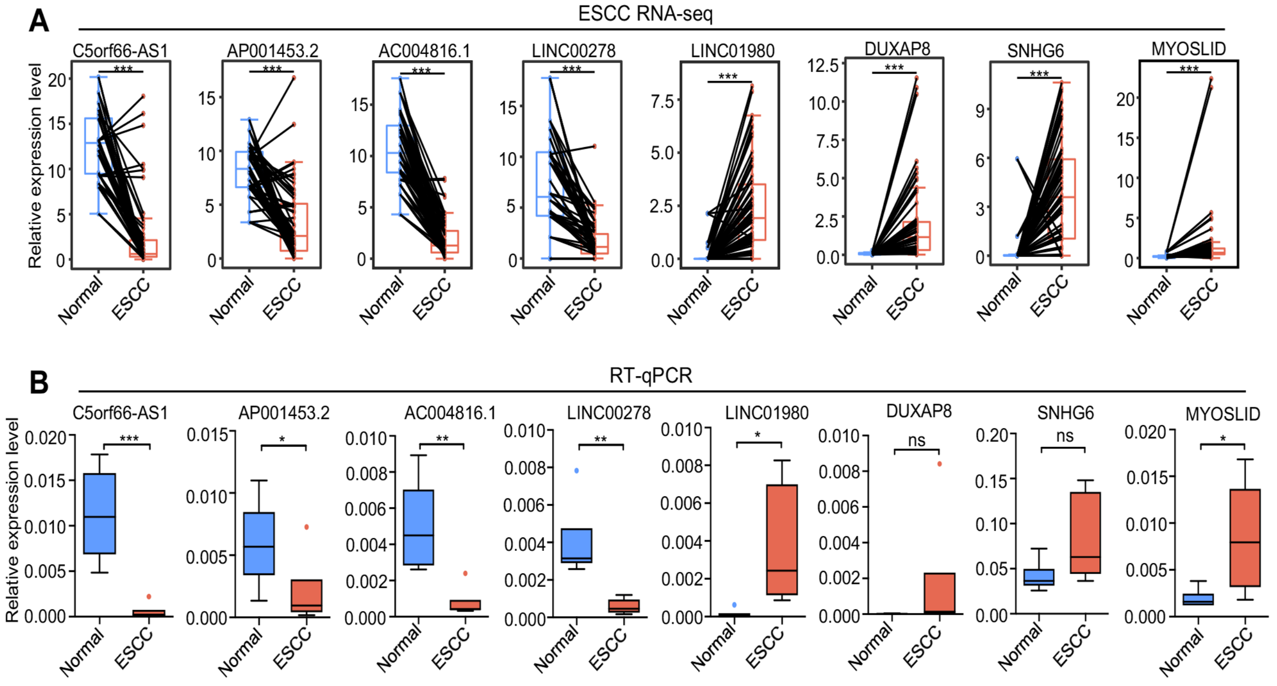

Supplementary Figure 1. Differentially expressed lncRNAs between ESCC tumors and normal tissues, related to Figure 1. (A) Displays of differential expressed lncRNAs from our high-throughput RNA-Seq data. (B) RT-qPCR analyses showing lncRNA expression levels between ESCC tumors and normal tissues. LncRNA expression is normalized to *ACTB* mRNA level.

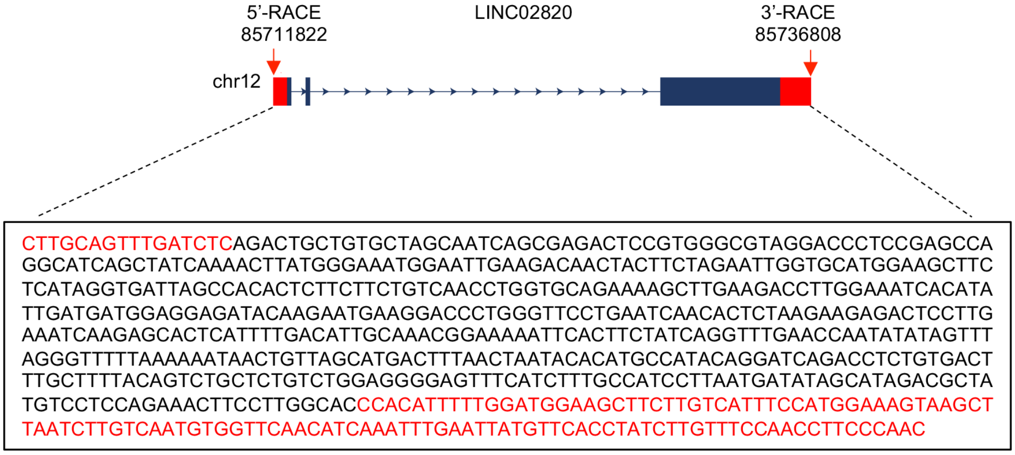

Supplementary Figure 2. Sequence of the full length LINC02820 variant in ESCC cells, related to Figure 2. Schematic representation of ENST00000555596 (depicted in dark blue) and LINC02820 we identified, consisting of three exons. The black-color sequence corresponds to ENST00000555596, while the red-color sequence represents the additional sequence of LINC02820.

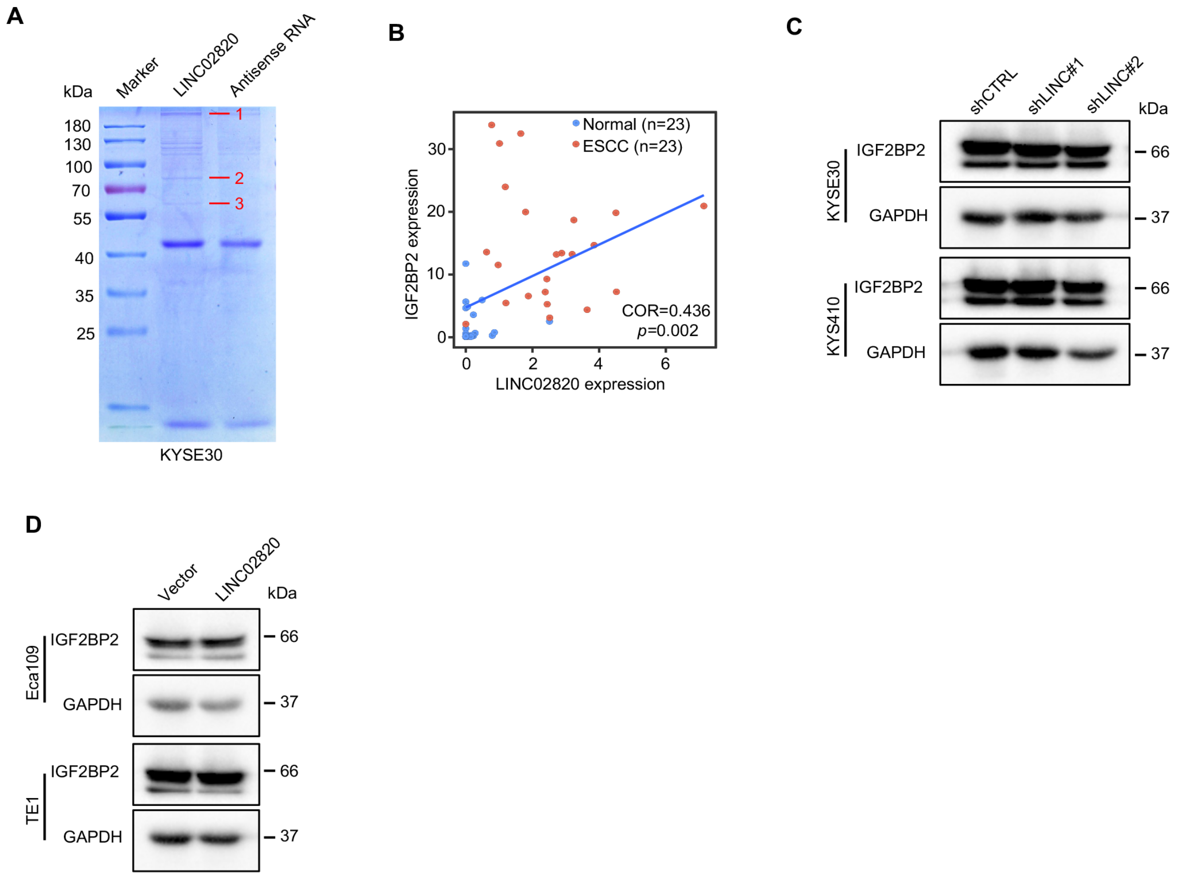

Supplementary Figure 3. IGF2BP2 enhances LINC02820 stability in ESCC cells, related to Figure 5. (A) RNA pull-down assays and Coomassie blue staining of LINC02820-interacting proteins by the biotinylated LINC02820 or antisense transcripts in KYSE30 cell. (B) Pearson correlation analysis of LINC02820 and *IGF2BP2* mRNA expression in GSE130078 dataset. (C, D) Immunoblot analyses of IGF2BP2 expression level in LINC02820 knockdown (C) and overexpression (D) ESCC cells.

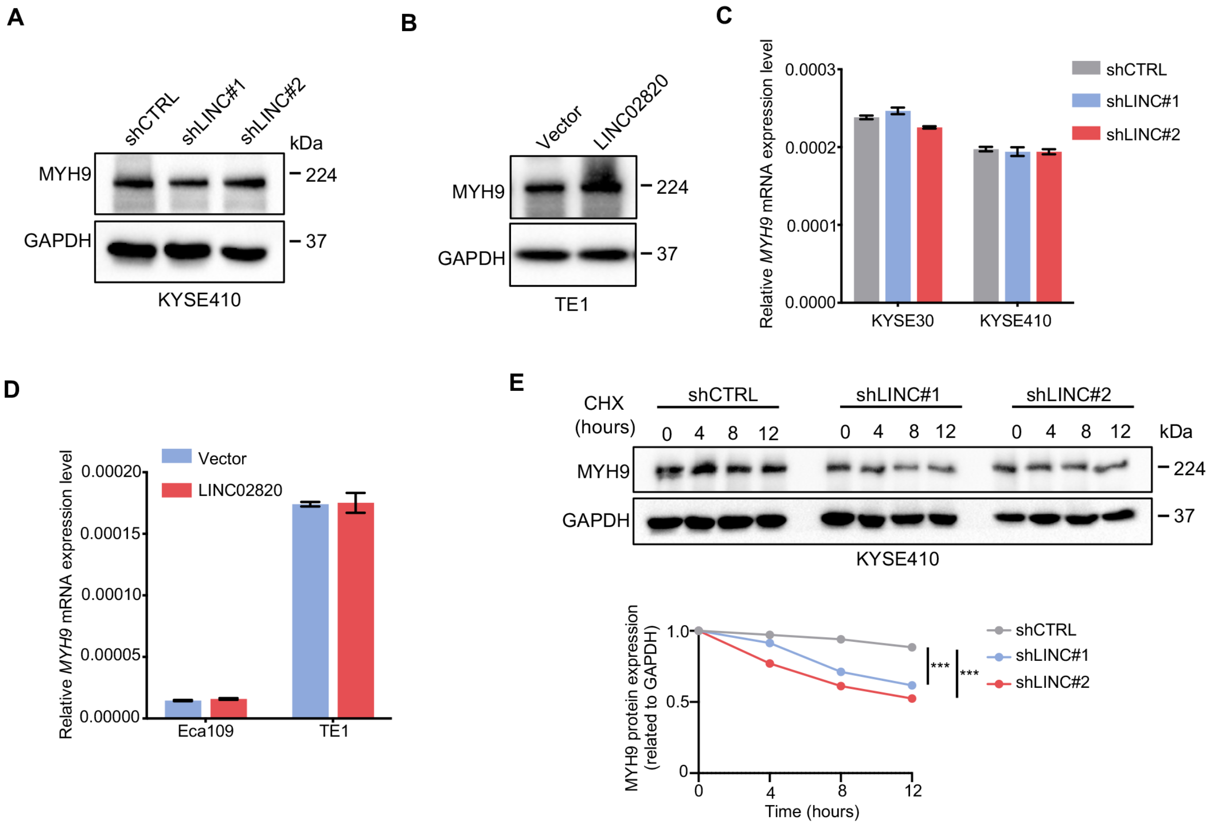

Supplementary Figure 4. LINC02820 binds to and increases MYH9 protein in ESCC cells, related to Figure 6. (A, B), RT-qPCR analysis of *MYH9* mRNA expression in LINC02820 knockdown (A) and overexpression (B) ESCC cells. LINC02820 expression is normalized to *GAPDH* mRNA. (C, D), Immunoblot analyses of MYH9 expression in LINC02820 knockdown (C) and overexpression (D) ESCC cells. (E) Immunoblot analyses of MYH9 levels in different KYSE410 cells treated with cycloheximide for indicated times.
